# Management of frailty: a protocol of a network meta-analysis of randomized controlled trials

**DOI:** 10.1186/s13643-017-0522-7

**Published:** 2017-07-05

**Authors:** Ahmed M. Negm, Courtney C. Kennedy, Lehana Thabane, Areti-Angeliki Veroniki, Jonathan D. Adachi, Julie Richardson, Ian D. Cameron, Aidan Giangregorio, Alexandra Papaioannou

**Affiliations:** 1 Geriatric Education and Research in Aging Sciences (GERAS) Centre, St Peter’s Hospital, 88 Maplewood Avenue, Hamilton, ON L8M 1W9 Canada; 2 School of Rehabilitation Science, McMaster University, 1400 Main Street West, Hamilton, ON L8S 1C7A Canada; 3 Department of Health Research Methods, Evidence, and Impact, McMaster University, 1280 Main St West, Hamilton, ON L8S 4L8 Canada; 4 Li Ka Shing Knowledge Institute, St. Michael’s Hospital, 209 Victoria Street, East Building, Toronto, Ontario M5B 1W8 Canada; 5 Department of Medicine, McMaster University, 1280 Main St West, Hamilton, ON L8S 4L8 Canada; 6 John Walsh Centre for Rehabilitation Research, Sydney Medical School Northern, University of Sydney, Kolling Institute, Royal North Shore Hospital, St Leonards, NSW 2065 Australia

**Keywords:** Frail elderly, Functional capacity, Systematic review, Randomized controlled trial, Network meta-analysis, Intervention, Protocol, Frailty

## Abstract

**Background:**

Frailty is a common syndrome affecting 5–17% of community-dwelling older adults. Various interventions are used to prevent or treat frailty. Given the diversity of singular and multi-faceted frailty interventions, not all of them have been compared in head-to-head studies. Network meta-analyses provide an approach to simultaneous consideration of the relative effectiveness of multiple treatment alternatives. This systematic review and network meta-analysis of RCTs aims to determine the comparative effect of interventions targeting the prevention or treatment of frailty.

**Method:**

We will identify relevant RCTs, in any language and publication date, by a systematic search of databases including MEDLINE, EMBASE, CINAHL, AMED, the Cochrane Central Registry of Controlled Trials (CENTRAL), HealthSTAR, DARE, PsychINFO, PEDro, SCOPUS, and Scielo. Duplicate title and abstract and full-text screening will be performed. Authors will extract data and assess risk of bias (using the Cochrane Risk of Bias tool) of eligible studies. The review interventions will include (1) physical activity only, (2) physical activity with protein supplementation or other nutritional supplementation, (3) psychosocial intervention, (4) medication management, (5) pharmacotherapy, and (6) multi-faceted intervention (defined as an intervention that combine physical activity and/or nutrition with any of the following: (1) psychosocial intervention, (2) medication management, and (3) pharmacotherapy). Our primary outcome is difference in change of physical frailty from baseline measured by a reliable and valid frailty measure. Secondary outcomes and the assessments are (1) cognition, (2) short physical performance battery, (3) any other physical performance measure, (4) treatment cost, (5) quality of life, and (6) any adverse outcome. We will conduct a network meta-analysis using a Bayesian hierarchical model. We will also estimate the ranking probabilities for all treatments at each possible rank for each intervention and will assess the certainty of the estimates of effect using GRADE approach.

**Discussion:**

To the best of our knowledge, this will be the first systematic review and network meta-analysis considering the direct and indirect effect of interventions targeting frailty prevention or treatment. Given the established high prevalence and socio-economic burden of frailty, there is an urgent need for a high-quality systematic review to inform evidence-based management of frailty.

**Systematic review registration:**

PROSPERO 2016 CRD42016037465.

**Electronic supplementary material:**

The online version of this article (doi:10.1186/s13643-017-0522-7) contains supplementary material, which is available to authorized users.

## Background

Frailty is defined as a clinical condition with increased vulnerability, which results from aging-related degeneration across psychological, physical, and social functioning [1, 2]. It is a common syndrome occurring in 5–17% of community-dwelling older adults [3]. The prevalence of frailty increases to more than 32% in persons aged over 90 years [4], and it is expected to continue to increase as the population ages [5, 6]. Individuals who are frail have a 1.2- to 2.5-fold increase in the risk of falls, institutionalization, and mortality [7]. Frailty affects quality of life, morbidity, and mortality and results in considerable medical and public spending expense [8] such that it is now seen as one of the major challenges for health services. Effective interventions are needed to manage and decrease burden of frailty on older adults and their families/caregivers.

Results of frailty management studies showed contradicting evidence; for example, previous frailty intervention studies used comprehensive geriatric assessment [9, 10] and rehabilitation intervention models [11] showed effectiveness in improving physical function. In contrast, other studies used the same approach (comprehensive geriatric assessment) in the same population and did not show significant improvement in physical function [12, 13]. Two recent randomized-controlled trials (RCTs) applied multifactorial interdisciplinary intervention [14] and showed effectiveness in reducing frailty [15, 16]. Other systematic reviews examined individual interventions targeting frailty such as exercise [17, 18] and home-based support [19] and showed beneficial effects as well. There are also a few ongoing frailty intervention trials that will be completed within the next few months [20, 21].

Since RCTs and previous traditional meta-analyses evaluated only the relative efficacy of two frailty interventions at a time, the relative effects of different frailty interventions are not well understood. Given the diversity of singular and multi-faceted interventions addressing frailty, not all of them have been compared in head-to-head studies. New methodological techniques are required to provide effect estimates for all comparisons. Network meta-analyses provide an approach to a simultaneous consideration of the relative effectiveness of multiple treatment alternatives [22, 23]. Due to the mixed evidence from the frailty intervention studies, a systematic review and network meta-analysis is needed to incorporate the recent studies to the current evidence of frailty intervention and compare the effectiveness of individual versus multimodal frailty interventions. Synthesizing the current evidence of frailty interventions will enable researchers, clinicians, and policy makers to determine the effectiveness of the current frailty interventions. We will combine direct (i.e., head-to-head trials) and indirect comparisons (which provides the relative treatment effects between two treatments when head-to-head trials are not available [24]) using a network meta-analysis [25]. Therefore, we will conduct a network meta-analysis of RCTs to determine the comparative effectiveness of interventions targeting the prevention or treatment of frailty in older adults. We aim to examine all types of interventions targeting frailty including comprehensive geriatric assessment, physical activity, nutrition, psychosocial intervention, pharmacotherapy, medication management, or multimodal interventions.

## Methods/design

This review will conform to the Preferred Reporting Items for The PRISMA Extension Statement for Reporting of Systematic Reviews Incorporating Network Meta-Analyses of Health Care Interventions (see Additional file 1 shows the PRISMA-P checklist) [26]. This protocol is registered in PROSPERO, systematic review registration: PROSPERO 2016 CRD42016037465.

### Search strategy

We will identify relevant RCTs, in any language and publication date, by a systematic search of MEDLINE, EMBASE, CINAHL, AMED, the Cochrane Central Registry of Controlled Trials (CENTRAL), HealthSTAR, DARE, PsychINFO, PEDro, SCOPUS, and Scielo from the inception of each database. An experienced librarian will be involved in designing our search strategies in individual databases (see Additional file 2 shows search strategies in the included databases).

The search strategy will combine text terms describing frailty with terms describing multi-faceted or singular interventions. We will scan the reference lists of all included trials and relevant reviews. Also, the authors of this review who are leaders in the frailty field will identify publications about frailty interventions. We will search three clinical trial registries to identify ongoing trials: Clinical Trials Registry, Current Controlled Trials, and the World Health Organization International Clinical Trials Registry Platform. We will search unpublished work using key meeting proceedings and the following websites: (1) ProQuest Dissertations and Theses, (2) E-Thos, and (3) OpenGrey.

### Eligibility criteria

Studies will be included if (1) one or more interventions (described below) were applied; (2) comparator was a control, usual care, or another intervention; (3) primary or secondary outcome was frailty or physical function change (using frailty measure or any other physical performance measure); (4) the study is full-text English RCT; and (5) the study includes only adults.

### Definition of interventions

Based on our preliminary search and clinical judgment of this review authors, the included interventions will be (1) physical activity intervention program only, (2) physical activity program with protein supplementation or other nutritional supplementation, (3) psychosocial intervention only, (4) medication management (such as reducing poly-pharmacy), (5) pharmacotherapy (such as sarcopenic medication or hormone therapy), and (6) multi-faceted intervention (defined as an intervention that combines physical activity and/or nutrition with any of the following: (1) psychosocial intervention, (2) medication management, and (3) pharmacotherapy). Relevant analyses will most likely include a seven-node network meta-analysis (including a control node).

### Types of outcome

The primary outcome will be difference in change of physical frailty from baseline measured by a reliable and valid frailty measure or physical performance measure when used as a surrogate for frailty measure [27]. If a study included both frailty and physical performance measure, the frailty measure will be included in the analysis. Secondary outcomes will include (1) cognition, which includes any measure of cognitive functions (such as memory, attention, language, and executive function); (2) short physical performance battery (SPPB), which is composed of three assessments and each assessment score between 0 and 4. A final summary performance score out of 12 is calculated, with higher scores indicating superior lower extremity function [27]. The SPPB has also been validated and has demonstrated good internal consistency [27]; (3) any other physical performance measure; (4) treatment cost; (5) quality of life; and (6) any Adverse outcome.

### Study selection

Using a standard form, the eligibility assessment of title and abstract of citations obtained from the search will be performed by two independent reviewers unblinded to author, journal, and country. The study form will be pilot-tested by the review team. Any disagreements will be resolved through consensus or with assistance from a third author if necessary. After title and abstract screening for potentially eligible studies, two reviewers will use a standard form to check the full-text articles for eligibility independently and any disagreements will be resolved through consensus or with assistance from a third author if necessary. The agreement between the two reviewers (on the title and abstract and full-text selection) will be assessed by examining raw agreement and unweighted kappa (*k*). The agreement between reviewers will be interpreted using the following thresholds: ≤0 as poor agreement, 0.01–0.20 as slight agreement, 0.21–0.40 as fair agreement, 0.41–0.60 as moderate agreement, 0.61–0.80 as substantial agreement, and >0.80 as almost perfect agreement [28].

### Data extraction and management

A data extraction form will be developed for this review and pilot tested independently on two randomly selected studies by two reviewers to ensure consistency in extraction. The extraction form will be refined accordingly and data will be extracted in duplicate. The extracted information will include the characteristics of participants (age, gender, frailty severity, and method of diagnosis), types and characteristics of intervention (frequency, descriptions, durations), and all reported outcome measures, baseline data, and post-treatment data points. At each data point, we will extract (1) mean or mean change from the baseline and standard deviations (SDs) or the information from which SD could be derived, such as standard error or confidence interval (CI) for continuous outcomes; (2) number of events and total number of patients per arm or odds ratio with a measure of uncertainty such as a standard error, 95% CI, or an exact *P* value for dichotomous data; and (3) counts and total number of patients per arm or rate ratio with a measure of uncertainty such as a standard error, 95% CI, or an exact *P* value for count outcomes. If a trial presents outcomes at more than one time point, data for all time points will be extracted; however, only data acquired immediately post-treatment and 1 year follow-up (or the closest time point) will be used in the meta-analysis.

### Assessment of risk of bias in included studies

The risk of bias of included trials will be assessed using the modified version of Cochrane’s tool for assessing risk of bias [29, 30]. The following domains are assessed according to this tool:

1. Sequence generation (selection bias)
2. Allocation concealment (selection bias)
3. Blinding of participants and personnel (performance bias)
4. Blinding of outcome assessment (detection bias)
5. Incomplete outcome data (attrition bias)
6. Selective outcome reporting (reporting bias)
7. Other potential sources of bias (including for-profit bias)

Each of the domains will be judged as “definitely yes,” “probably yes,” “probably no,” and “definitely no” for each of the domains, with “definitely yes” and “probably yes” ultimately assigned low risk of bias and “definitely no” and “probably no” assigned high risk of bias [29, 30]. We will summarize the risk of bias judgments across different studies for each of the domains listed. Any disagreements regarding risk of bias will be resolved by consensus or with assistance from a third author if necessary.

## Data synthesis

### Network geometry

Qualitative description of network geometry will be provided and accompanied by a network plot [31]. We will obtain a network plot to assess if the trial treatments are connected. We will evaluate the quantitative metrics assessing features of network geometry such as diversity (number of treatments and how frequent they are examined) and co-occurrence (whether certain treatment comparisons are more or less common and the extent of comparisons between different treatments) [31].

### Measures of treatment effect

For dichotomous outcomes, we will calculate the odds ratio with a 95% credible interval [32]. For continuous outcomes, we will calculate the mean difference with a 95% credible interval. We will use the standardized mean difference with a 95% credible interval if the included trials use different scales for a continuous outcome. In case the same outcome is described by both dichotomous and continuous data from different studies, we will convert mean differences or standardized mean differences to odds ratio estimates [29]. For count outcomes, such as the number of adverse events, we will calculate the rate ratio with a 95% credible interval. For multi-arm studies, we will use the data from all reported comparisons.

### Dealing with missing data

We will contact study authors to obtain missing data. Where this is not possible or missing data could lead to serious biases, we will explore the impact of including these studies in the overall assessment of results by a sensitivity analysis for continuous and binary data. If numerical outcome data such as SDs or correlation coefficients are missing and they cannot be obtained from the study authors, we will calculate them from other available statistics such as *P* values, according to the methods described in the Cochrane Handbook for Systematic Reviews of Interventions [29]. In case outcome values are reported without a measure of variance, SDs will be imputed according to the method suggested by Furukawa et al. [33]. We will report information regarding loss to follow-up and we will assess this as a potential risk of bias. We will perform an intention-to-treat analysis whenever possible. Otherwise, we will use the data that are available to us (e.g., a trial may have reported only “per-protocol” analysis results).

## Assessment of transitivity across treatment comparisons

We will assess the assumption of transitivity by comparing the distribution of the potential effect modifiers (which include (1) baseline frailty level, (2) age, (3) sex, and (4) trials with low risk of bias compared to trials with high risk of bias, across the different pairwise comparisons) to ensure that they are on average balanced. Control groups (e.g., standard care or placebo) will be assessed for their similarity across treatment comparisons [34].

## Methods for direct and indirect or mixed treatment comparisons

We will conduct network meta-analyses to compare multiple interventions simultaneously for each of the primary and secondary outcomes. We will perform a network meta-analysis of trials in which participants will be reasonably similar (i.e., there will be no major concerns about transitivity assumption). We will conduct a network meta-analysis using a Bayesian hierarchical model, implemented by the gemtc package in R [35]. We will model the treatment contrast (i.e., mean difference or standardized mean difference for continuous outcomes, log odds ratio for dichotomous outcomes, rate ratio for count outcomes) for any two interventions as a function of comparisons between each individual intervention. The reference group will be usual care or control [36]. We will use a hierarchical Bayesian model using a non-informative prior for the treatment effect parameter and between-trial variance due to lack of previous evidence of frailty intervention [37, 38]. Considering the expected heterogeneity of the included studies, we will use a random-effects model. Model convergence will be assessed using established methods including Gelman-Rubin diagnostics and inspection of Monte Carlo errors [36].

### Relative treatment ranking

We will also estimate the ranking probabilities for all treatments at each possible rank for each intervention. Then, we will obtain the treatment hierarchy using the surface under the cumulative ranking (SUCRA) curve and mean ranks [39]. SUCRA can also be expressed as a percentage of a treatment that can be ranked first without uncertainty. We will use the rank-heat plot to visually present the treatment hierarchy across the multiple outcomes of this review [40].

## Assessment of statistical heterogeneity and inconsistency

In each network meta-analysis, we will assume a common estimate for the heterogeneity variance across the different comparisons [41] since we expect that the heterogeneity will be similar across treatment comparisons. The assessment of statistical heterogeneity in the entire network will be based on the magnitude of the heterogeneity variance parameter estimated from the network meta-analysis models.

To check the assumption of consistency in the entire network, we will use the design-by-treatment interaction model [29, 42]. This method accounts for different sources of inconsistency that can occur when studies with different designs (two-arm trials versus three-arm trials) give different results and when there is disagreement between direct and indirect evidence. Using this approach, we will make inferences about the presence of inconsistency from any source in the entire network based on a chi^2^ test. If the design-by-treatment interaction model shows evidence of inconsistency, we will use the loop-specific approach [43] (if we have a network with at least one closed loop) to detect the paths of the network that are responsible of inconsistency locally. This method evaluates the consistency assumption in each closed loop of the network separately as the difference between direct and indirect estimates for a specific comparison in the loop (inconsistency factor) [34]. Then, the magnitude of the inconsistency factors and their 95% CIs can be used to make inferences about inconsistency in each loop and its statistical significance. We will assume common heterogeneity estimate within each loop, and the restricted maximum likelihood method will be used [44].

### Subgroup and meta-regression analysis

If sufficient studies are available, we will perform subgroup analyses using possible sources of inconsistency or heterogeneity between studies such as age, gender, educational level, and comorbidity. Our a priori hypothesis will be older, female, lower educational level, and more comorbidity subgroups may show less improvement in the primary and secondary outcomes. We will conduct additional meta-regression analyses using random-effects network meta-regression models to examine potential effect moderators such as the mean age of participants, baseline frailty level, adherence level to treatment, and the frailty measure.

### Sensitivity analysis

If sufficient studies are available, we will assess the effect of excluding (1) studies with high risk of bias, (2) studies with missing data, and (3) studies with imputed data (to ensure that our imputations do not bias our network meta-analysis results) from the analyses.

## Certainty of the evidence and summary of findings table

We will use the GRADE (Grading of Recommendations Assessment, Development and Evaluation) approach of network meta-analysis [45] to assess the certainty of direct, indirect, and mixed network meta-analysis effect estimates for each outcome. The certainty of evidence of direct effect estimates for each outcome will be rated as high, moderate, low, or very low using the GRADE rating system [46]. In the GRADE system, RCTs start as high-quality evidence but may be rated down due to limitation in study design, inconsistency, imprecision, indirectness, and publication bias [30, 47].

The indirect effect estimate will be calculated from the available loops of evidence (including loops with a single common comparator (first order) or more than one intervening treatment (higher orders) connecting the two interventions of the comparison of interest). The quality of indirect evidence will focus on the dominant first-order loop (loops with a single common comparator connecting the two interventions of the comparison of interest). The quality of evidence rating for indirect comparisons will be the lower of the ratings of quality for the two direct estimates that contribute to the first-order loop of the indirect comparison. For instance, if one of the direct comparisons will be rated as low and the other will be rated as moderate evidence, we will rate the quality of indirect evidence as low [45]. We will rate down the quality of the indirect comparison one further level for violation of the transitivity assumption (similarity of trials in terms of population, intervention (type and dosing frequency), settings, and trial methodology) [45].

If both direct and indirect evidence are available, the network meta-analysis mixed estimate quality rating will come from the higher quality of the two. We will consider similarity between direct and indirect effect estimates (coherence) in our final quality rating. We will rate down the quality of the mixed network meta-analysis effect if there is incoherence between direct and indirect effect estimates (measured by the difference of point estimates and the extent of overlap of CIs and of direct and indirect effect estimates).

## Assessment of publication biases

For each treatment comparison, we will visually assess publication bias and small-study effects using funnel plots (using study’s effect estimates for the primary outcomes against their standard errors) [48, 49]. In the network, we will use a comparison-adjusted funnel plot to assess network-wide publication bias. We will chronologically order the treatments (from the oldest to the newest) [50]. Funnel plots will be drawn only when the number of studies is ≥10 (27). Funnel plot asymmetry might be due to publication bias but other reasons such as true heterogeneity are also possible.

Two authors will assess the quality criteria independently. Disagreements will be arbitrated by a third author until we reach consensus. The main results of the review will be presented in a summary of findings (SoF) table [51]. The SoF table will include an overall grading of the quality of evidence related to each of the comparisons, using the GRADE approach [52].

## Discussion

Given the established high prevalence and socio-economic burden of frailty in aging population, and the paucity of evidence on the comparative effectiveness of treatment options, there is a critical need for a high-quality systematic review to inform evidence-based management of frailty.

To the best of our knowledge to date, there is no systematic review and network meta-analysis considering the direct and indirect effect of interventions targeting frailty prevention or treatment. This analysis will include a comparison of several different prevention/treatment options including singular (e.g., physical activity or nutrition) and multi-faceted interventions. Methodologically, our review has several strengths including (1) covering articles up to the present date which is an important consideration given the recent focus on interventions for frailty; (2) exploring a wider range of literature databases than previous reviews and including eligible articles in all languages; (3) determining trial eligibility and collecting data will be made in teams of reviewers, independently and in duplicate; (4) using GRADE approach to evaluate our confidence in treatment effects and present our findings with GRADE SoF tables; and (5) meta-regression and subgroup analyses will be conducted, consistent with the best current practices.

Potential challenges and limitations of the proposed review include high heterogeneity, poor quality of reporting and/or methodological rigor in included trials, and difficulty in interpreting measures of effect when the pooled estimates come from trials that measured the outcome using different frailty tools and physical performance measures. Intervening to prevent or treat frailty is a relatively new field and whether the breadth of articles will be available to conduct comparisons is not known. Another likely limitation, unique to network meta-analyses, will be lack of available treatment comparisons to build robust networks for our analyses.

The findings of our review will inform clinicians and policy makers about evidence-based components, doses, and duration of interventions to prevent or alleviate frailty. There is currently consensus regarding the importance of screening for frailty and its adverse effects [14], but research evidence regarding how we treat or prevent frailty is lacking. This review will facilitate updating clinical practice guidelines of frailty management.

## Additional files

Additional file 1: The PRISMA-P checklist. (DOCX 35 kb)

Additional file 2: Search strategies performed in MEDLINE, EMBASE, CENTRAL, AMID, HealthStar, and PsychInfo. (DOCX 41 kb)

## Abbreviations

CI: Confidence interval
GRADE: Grading of Recommendations Assessment, Development and Evaluation
PRISMA: Preferred Reporting Items for Systematic Reviews and Meta-Analyses
RCT: Randomized-controlled trial
SD: Standard deviation
SoF: Summary of findings
SUCRA: Surface under the cumulative ranking
