# Supplementary material for: Management of frailty: a protocol of a network meta-analysis of randomized controlled trials
Source: Syst Rev. 2017 Jul 5;6:130. doi: 10.1186/s13643-017-0522-7 (PMC5499023; doi:10.1186/s13643-017-0522-7)
Supplement: Supplementary file 2 — Search strategies performed in MEDLINE, EMBASE, CENTRAL, AMID, HealthStar, and PsychInfo. (DOCX 41 kb) [file 13643_2017_522_MOESM2_ESM.docx]

**Additional file 1: Proposed search strategy for databases**

**HealthStar**

1. frail elderly.mp. or Frail Elderly/

2. frail*.ti,ab.

3. frailty.mp.

4. 1 or 2 or 3

5. limit 4 to (randomized controlled trial or "review")

6. random*.mp.

7. systematic review*.mp.

8. 6 or 7

9. 4 and 8

10. 5 or 9

**PsychInfo**

1. frail elderly.mp. or Frail Elderly/

2. frail*.ti,ab.

3. frailty.mp.

4. 1 or 2 or 3

5. random*.mp.

6. systematic review*.mp.

7. 5 or 6

8. 4 and 7

**AMID**

1. frail elderly.mp. or Frail Elderly/

2. frail*.ti,ab.

3. frailty.mp.

4. 1 or 2 or 3

5. random*.mp.

6. systematic review*.mp.

7. 5 or 6

8. 4 and 7

**Embase**

1. frail elderly.mp. or Frail Elderly/

2. frail*.ti,ab.

3. frailty.mp.

4. 1 or 2 or 3

5. limit 4 to randomized controlled trial

6. random*.mp.

7. systematic review*.mp.

8. 6 or 7

9. 4 and 8

10. 5 or 9

**MiDLine**

1. frail elderly.mp. or Frail Elderly/

2. frail*.ti,ab.

3. frailty.mp.

4. 1 or 2 or 3

5. limit 4 to (randomized controlled trial or systematic reviews)

6. random*.mp.

7. systematic review*.mp.

8. 6 or 7

9. 4 and 8

10. 5 or 9

**CENTRAL**

#1 Frail Elderly

#2 frail

#3 frailty

#4 random

#5 systematic review

#6 #4 or #5

#7 #1 or #2 or #3

#8 #6 and #7
